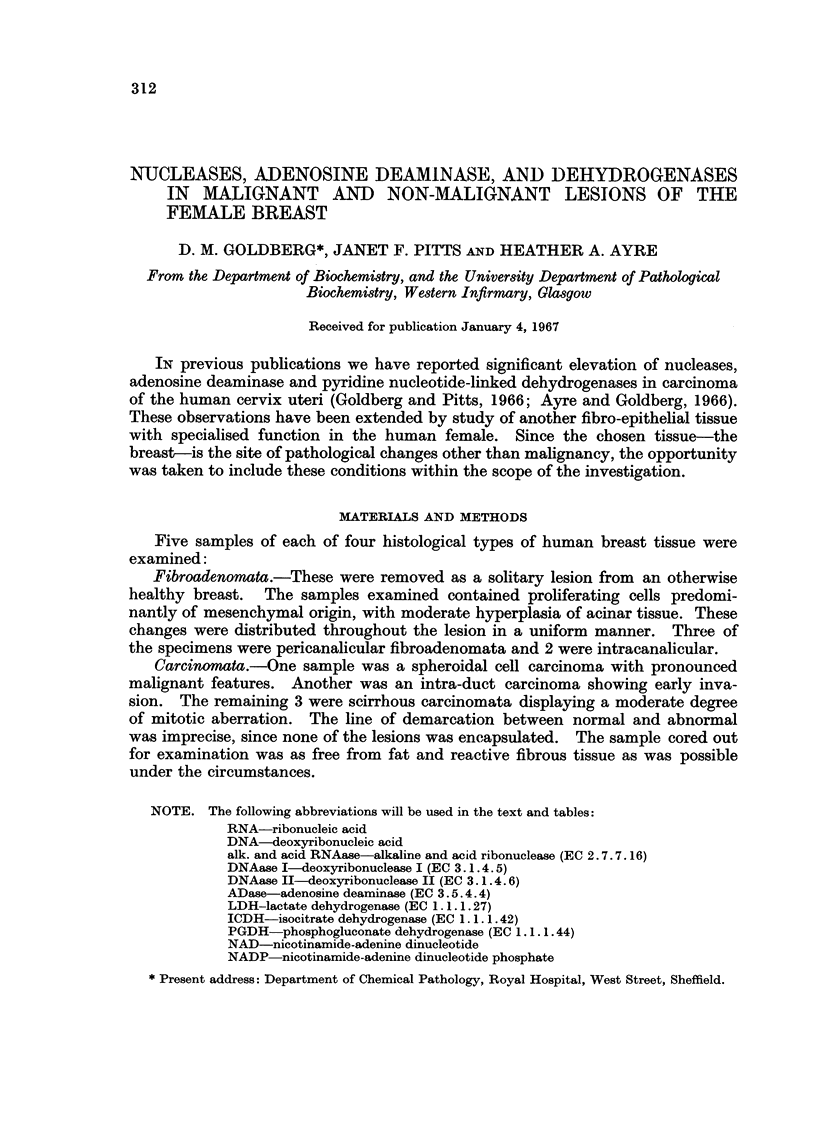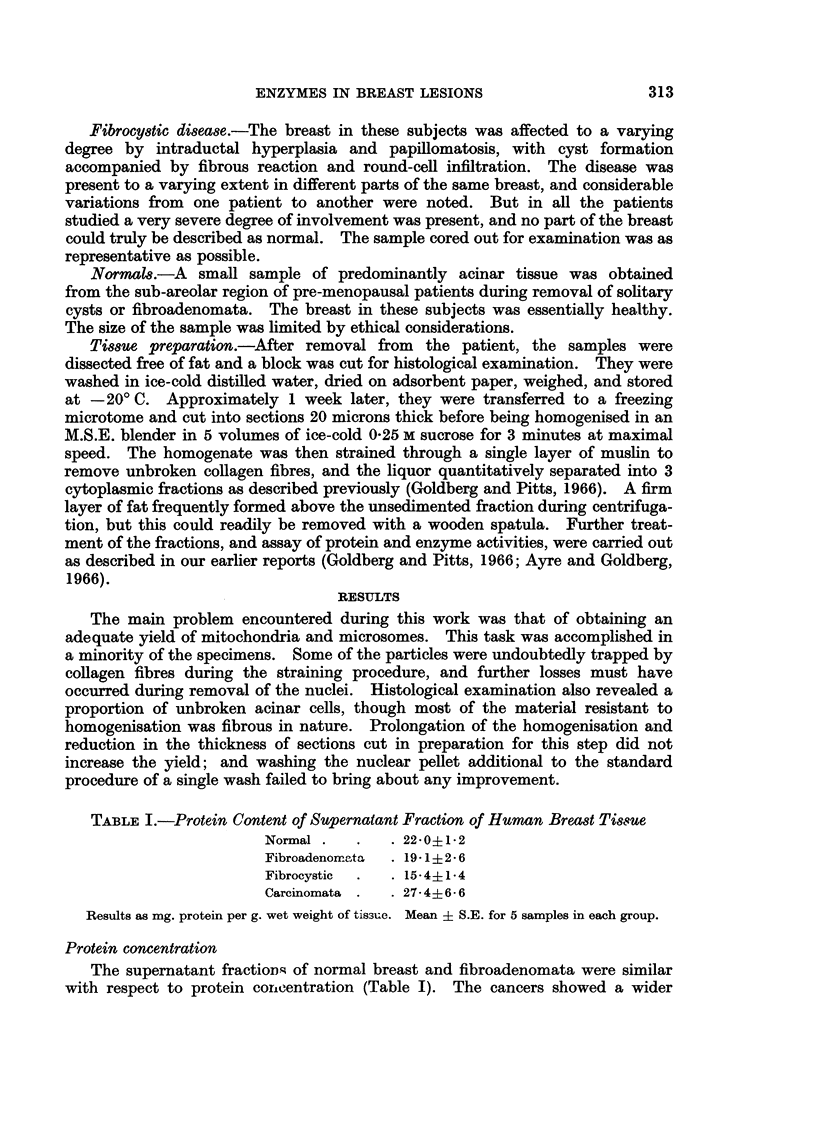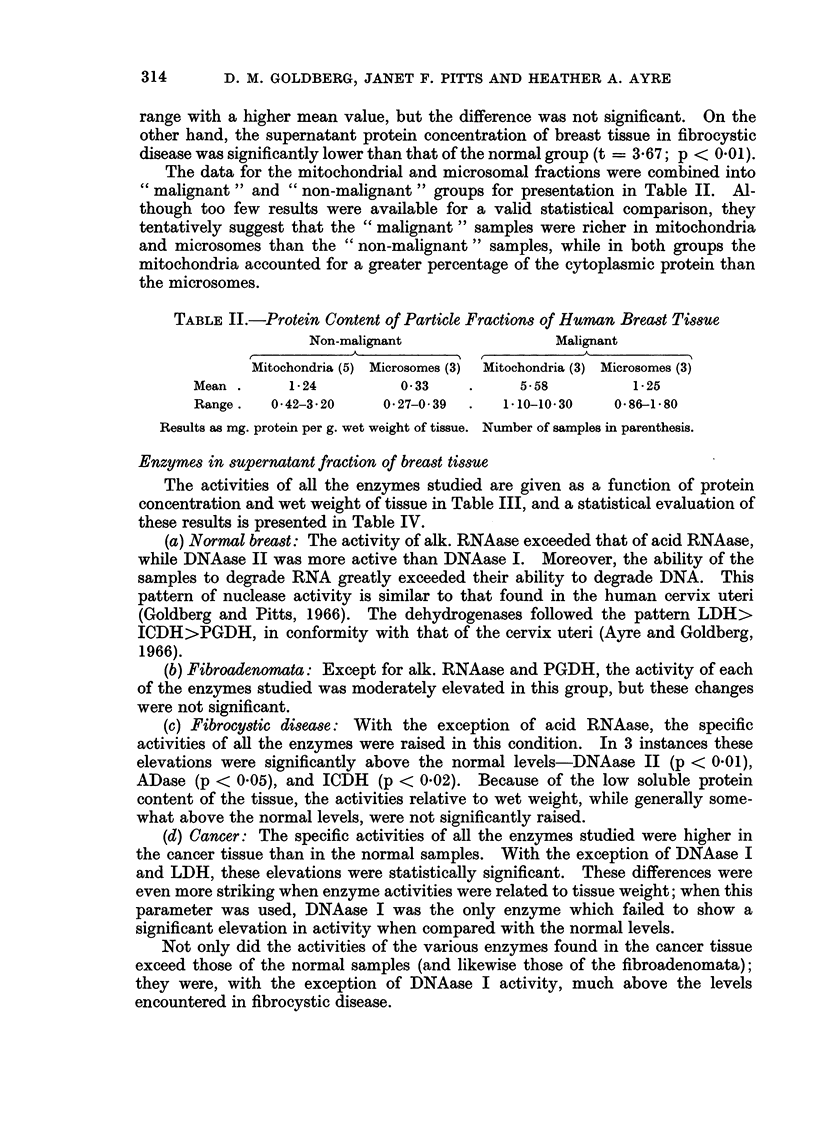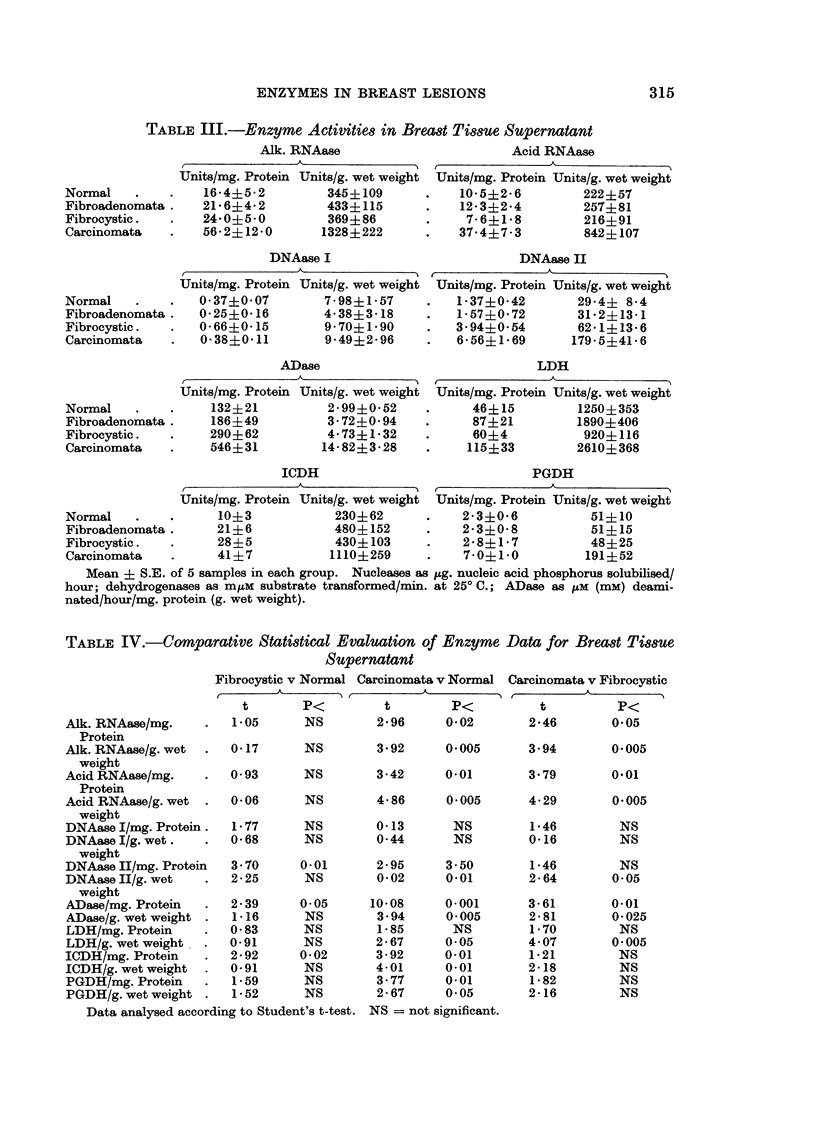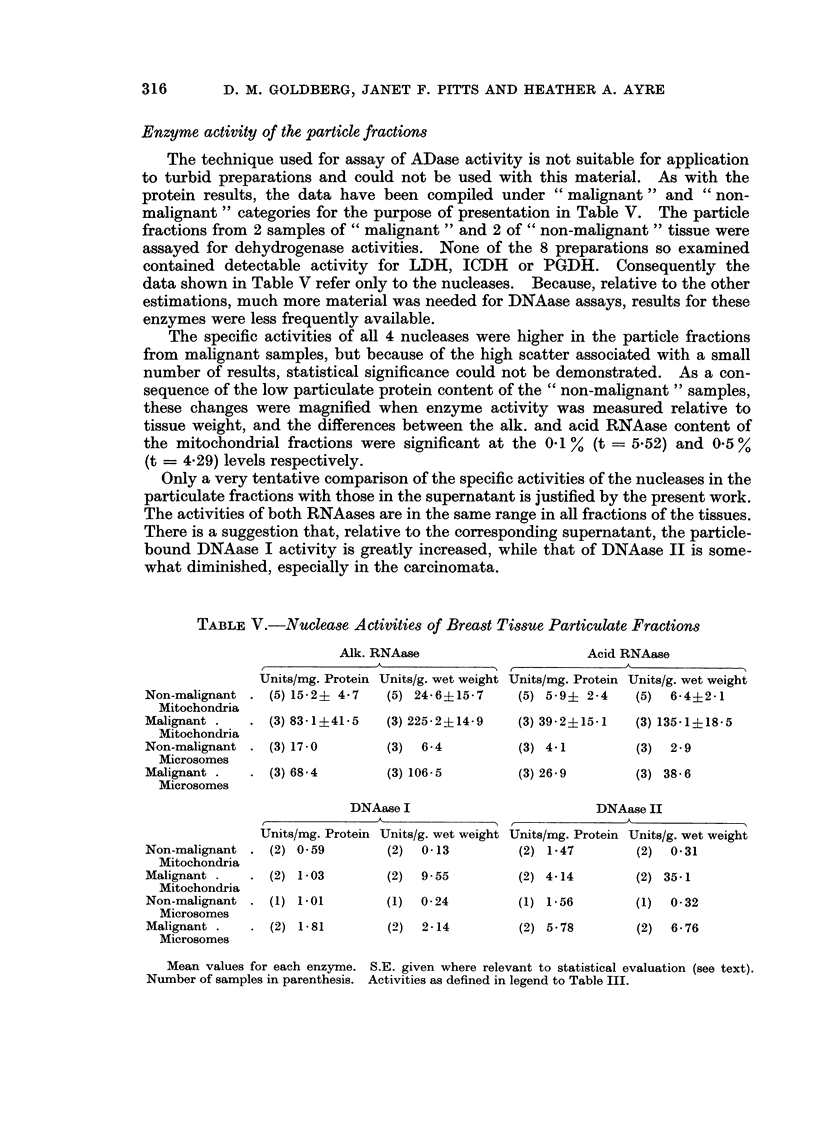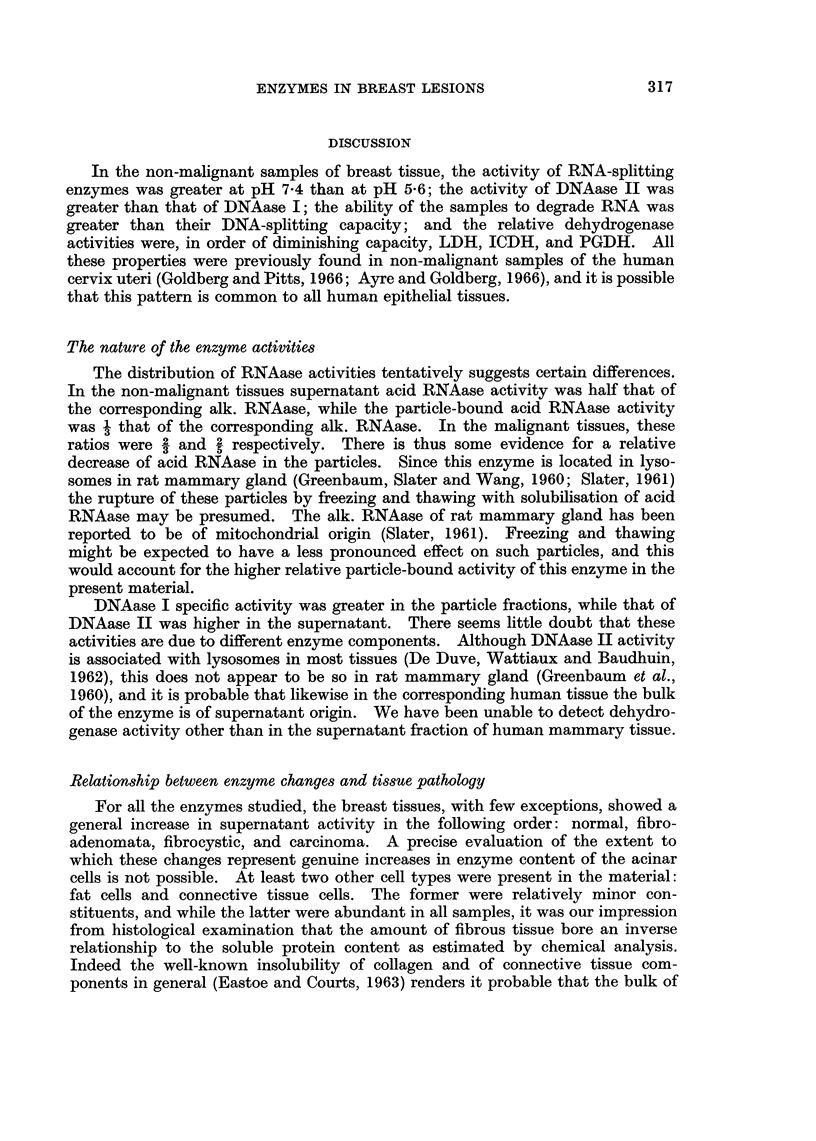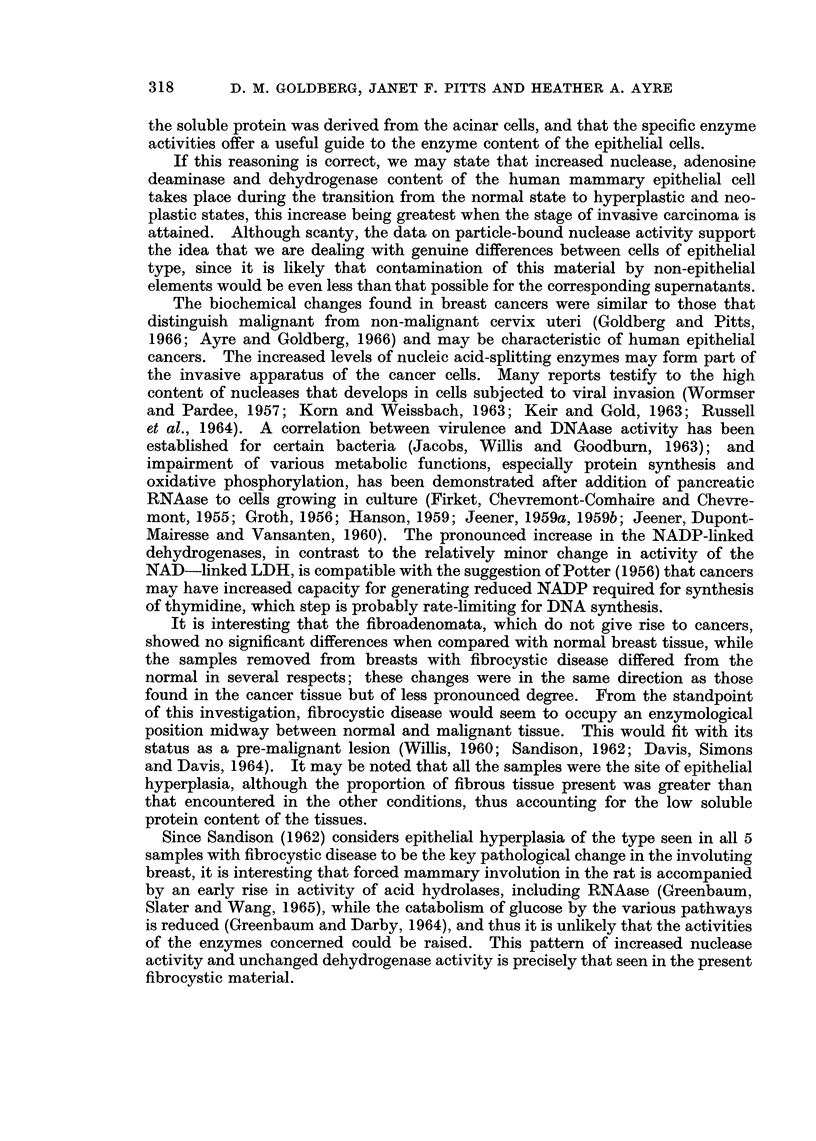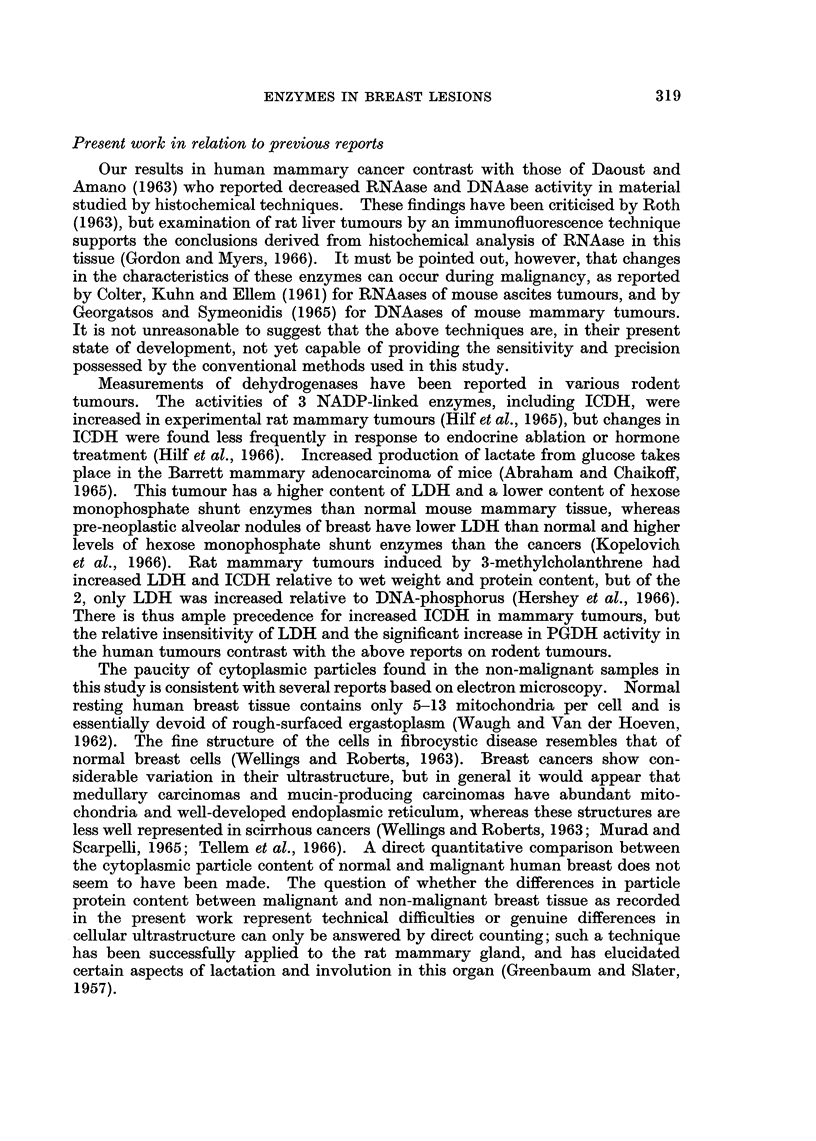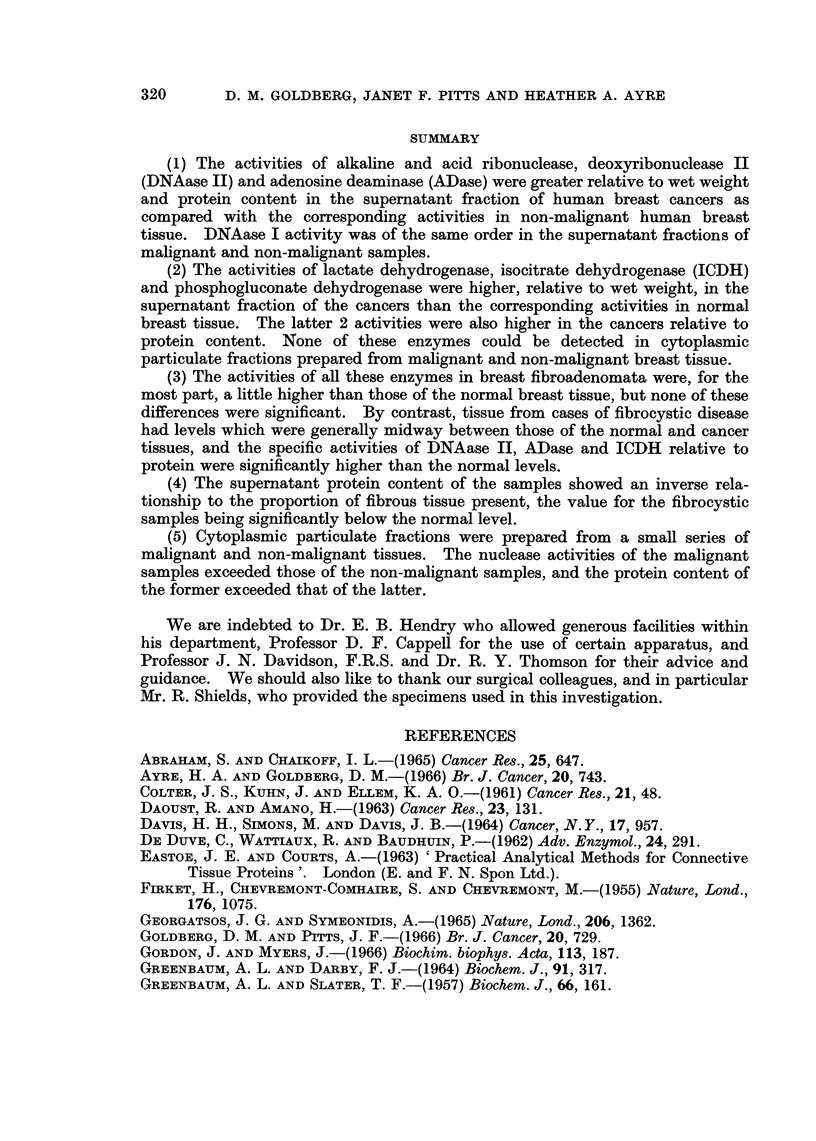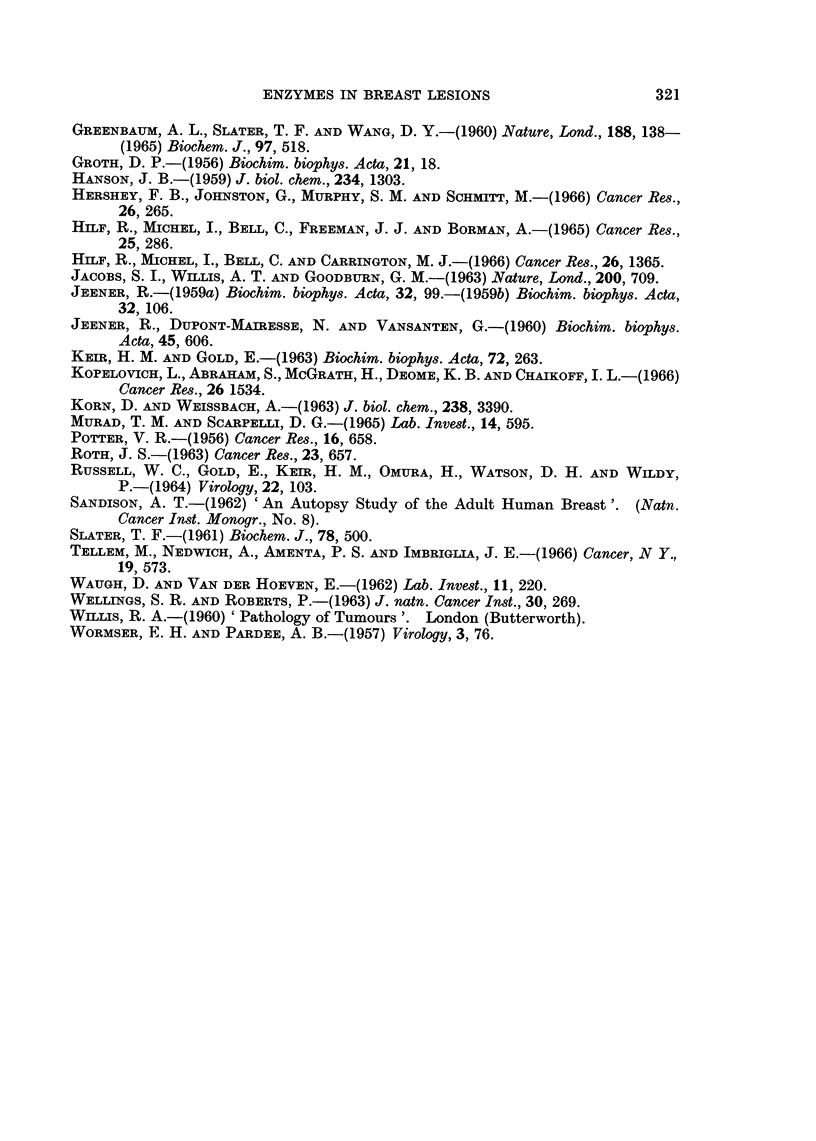# Nucleases, adenosine deaminase, and dehydrogenases in malignant and non-malignant lesions of the female breast.

**DOI:** 10.1038/bjc.1967.34

**Published:** 1967-06

**Authors:** D. M. Goldberg, J. F. Pitts, H. A. Ayre


### NUCLEASES, ADENOSINE DEAMINASE, AND DEHYDROGENASES IN MALIGNANT AND NON-MALIGNANT LESIONS OF THE FEMALE BREAST

D. M. GOLDBERG*, JANET F. PITTS AND HEATHER A. AYRE

*From the Department of Biochemistry, and the University Department of Pathological Biochemistry, Western Infirmary, Glasgow*

Received for publication January 4, 1967

IN previous publications we have reported significant elevation of nucleases, adenosine deaminase and pyridine nucleotide-linked dehydrogenases in carcinoma of the human cervix uteri (Goldberg and Pitts, 1966; Ayre and Goldberg, 1966). These observations have been extended by study of another fibro-epithelial tissue with specialised function in the human female. Since the chosen tissue—the breast—is the site of pathological changes other than malignancy, the opportunity was taken to include these conditions within the scope of the investigation.

### MATERIALS AND METHODS

Five samples of each of four histological types of human breast tissue were examined:

*Fibroadenomata*.—These were removed as a solitary lesion from an otherwise healthy breast. The samples examined contained proliferating cells predominantly of mesenchymal origin, with moderate hyperplasia of acinar tissue. These changes were distributed throughout the lesion in a uniform manner. Three of the specimens were pericanalicular fibroadenomata and 2 were intracanalicular.

*Carcinomata*.—One sample was a spheroidal cell carcinoma with pronounced malignant features. Another was an intra-duct carcinoma showing early invasion. The remaining 3 were scirrhous carcinomata displaying a moderate degree of mitotic aberration. The line of demarcation between normal and abnormal was imprecise, since none of the lesions was encapsulated. The sample cored out for examination was as free from fat and reactive fibrous tissue as was possible under the circumstances.

NOTE. The following abbreviations will be used in the text and tables:

RNA—ribonucleic acid
DNA—deoxyribonucleic acid
alk. and acid RNAase—alkaline and acid ribonuclease (EC 2.7.7.16)
DNAase I—deoxyribonuclease I (EC 3.1.4.5)
DNAase II—deoxyribonuclease II (EC 3.1.4.6)
ADase—adenosine deaminase (EC 3.5.4.4)
LDH-lactate dehydrogenase (EC 1.1.1.27)
ICDH—isocitrate dehydrogenase (EC 1.1.1.42)
PGDH—phosphogluconate dehydrogenase (EC 1.1.1.44)
NAD—nicotinamide-adenine dinucleotide
NADP—nicotinamide-adenine dinucleotide phosphate

* Present address: Department of Chemical Pathology, Royal Hospital, West Street, Sheffield.

*Fibrocystic disease.*—The breast in these subjects was affected to a varying degree by intraductal hyperplasia and papillomatosis, with cyst formation accompanied by fibrous reaction and round-cell infiltration. The disease was present to a varying extent in different parts of the same breast, and considerable variations from one patient to another were noted. But in all the patients studied a very severe degree of involvement was present, and no part of the breast could truly be described as normal. The sample cored out for examination was as representative as possible.

*Normals.*—A small sample of predominantly acinar tissue was obtained from the sub-areolar region of pre-menopausal patients during removal of solitary cysts or fibroadenomata. The breast in these subjects was essentially healthy. The size of the sample was limited by ethical considerations.

*Tissue preparation.*—After removal from the patient, the samples were dissected free of fat and a block was cut for histological examination. They were washed in ice-cold distilled water, dried on adsorbent paper, weighed, and stored at $-20°$ C. Approximately 1 week later, they were transferred to a freezing microtome and cut into sections 20 microns thick before being homogenised in an M.S.E. blender in 5 volumes of ice-cold 0·25 M sucrose for 3 minutes at maximal speed. The homogenate was then strained through a single layer of muslin to remove unbroken collagen fibres, and the liquor quantitatively separated into 3 cytoplasmic fractions as described previously (Goldberg and Pitts, 1966). A firm layer of fat frequently formed above the unsedimented fraction during centrifugation, but this could readily be removed with a wooden spatula. Further treatment of the fractions, and assay of protein and enzyme activities, were carried out as described in our earlier reports (Goldberg and Pitts, 1966; Ayre and Goldberg, 1966).

### RESULTS

The main problem encountered during this work was that of obtaining an adequate yield of mitochondria and microsomes. This task was accomplished in a minority of the specimens. Some of the particles were undoubtedly trapped by collagen fibres during the straining procedure, and further losses must have occurred during removal of the nuclei. Histological examination also revealed a proportion of unbroken acinar cells, though most of the material resistant to homogenisation was fibrous in nature. Prolongation of the homogenisation and reduction in the thickness of sections cut in preparation for this step did not increase the yield; and washing the nuclear pellet additional to the standard procedure of a single wash failed to bring about any improvement.

TABLE I.—*Protein Content of Supernatant Fraction of Human Breast Tissue*

| | |
|---|---|
| Normal | $22\cdot0 \pm 1\cdot2$ |
| Fibroadenomata | $19\cdot1 \pm 2\cdot6$ |
| Fibrocystic | $15\cdot4 \pm 1\cdot4$ |
| Carcinomata | $27\cdot4 \pm 6\cdot6$ |

Results as mg. protein per g. wet weight of tissue. Mean $\pm$ S.E. for 5 samples in each group.

### *Protein concentration*

The supernatant fractions of normal breast and fibroadenomata were similar with respect to protein concentration (Table I). The cancers showed a wider range with a higher mean value, but the difference was not significant. On the other hand, the supernatant protein concentration of breast tissue in fibrocystic disease was significantly lower than that of the normal group ($t = 3.67$; $p < 0.01$).

The data for the mitochondrial and microsomal fractions were combined into " malignant " and " non-malignant " groups for presentation in Table II. Although too few results were available for a valid statistical comparison, they tentatively suggest that the " malignant " samples were richer in mitochondria and microsomes than the " non-malignant " samples, while in both groups the mitochondria accounted for a greater percentage of the cytoplasmic protein than the microsomes.

TABLE II.—*Protein Content of Particle Fractions of Human Breast Tissue*

| | Non-malignant | | Malignant | |
|---|---|---|---|---|
| | Mitochondria (5) | Microsomes (3) | Mitochondria (3) | Microsomes (3) |
| Mean | 1·24 | 0·33 | 5·58 | 1·25 |
| Range | 0·42–3·20 | 0·27–0·39 | 1·10–10·30 | 0·86–1·80 |

Results as mg. protein per g. wet weight of tissue. Number of samples in parenthesis.

*Enzymes in supernatant fraction of breast tissue*

The activities of all the enzymes studied are given as a function of protein concentration and wet weight of tissue in Table III, and a statistical evaluation of these results is presented in Table IV.

(*a*) *Normal breast:* The activity of alk. RNAase exceeded that of acid RNAase, while DNAase II was more active than DNAase I. Moreover, the ability of the samples to degrade RNA greatly exceeded their ability to degrade DNA. This pattern of nuclease activity is similar to that found in the human cervix uteri (Goldberg and Pitts, 1966). The dehydrogenases followed the pattern LDH>ICDH>PGDH, in conformity with that of the cervix uteri (Ayre and Goldberg, 1966).

(*b*) *Fibroadenomata:* Except for alk. RNAase and PGDH, the activity of each of the enzymes studied was moderately elevated in this group, but these changes were not significant.

(*c*) *Fibrocystic disease:* With the exception of acid RNAase, the specific activities of all the enzymes were raised in this condition. In 3 instances these elevations were significantly above the normal levels—DNAase II ($p < 0.01$), ADase ($p < 0.05$), and ICDH ($p < 0.02$). Because of the low soluble protein content of the tissue, the activities relative to wet weight, while generally somewhat above the normal levels, were not significantly raised.

(*d*) *Cancer:* The specific activities of all the enzymes studied were higher in the cancer tissue than in the normal samples. With the exception of DNAase I and LDH, these elevations were statistically significant. These differences were even more striking when enzyme activities were related to tissue weight; when this parameter was used, DNAase I was the only enzyme which failed to show a significant elevation in activity when compared with the normal levels.

Not only did the activities of the various enzymes found in the cancer tissue exceed those of the normal samples (and likewise those of the fibroadenomata); they were, with the exception of DNAase I activity, much above the levels encountered in fibrocystic disease.

TABLE III.—*Enzyme Activities in Breast Tissue Supernatant*

| | Alk. RNAase | | Acid RNAase | |
|---|---|---|---|---|
| | Units/mg. Protein | Units/g. wet weight | Units/mg. Protein | Units/g. wet weight |
| Normal | 16·4±5·2 | 345±109 | 10·5±2·6 | 222±57 |
| Fibroadenomata | 21·6±4·2 | 433±115 | 12·3±2·4 | 257±81 |
| Fibrocystic | 24·0±5·0 | 369±86 | 7·6±1·8 | 216±91 |
| Carcinomata | 56·2±12·0 | 1328±222 | 37·4±7·3 | 842±107 |

| | DNAase I | | DNAase II | |
|---|---|---|---|---|
| | Units/mg. Protein | Units/g. wet weight | Units/mg. Protein | Units/g. wet weight |
| Normal | 0·37±0·07 | 7·98±1·57 | 1·37±0·42 | 29·4± 8·4 |
| Fibroadenomata | 0·25±0·16 | 4·38±3·18 | 1·57±0·72 | 31·2±13·1 |
| Fibrocystic | 0·66±0·15 | 9·70±1·90 | 3·94±0·54 | 62·1±13·6 |
| Carcinomata | 0·38±0·11 | 9·49±2·96 | 6·56±1·69 | 179·5±41·6 |

| | ADase | | LDH | |
|---|---|---|---|---|
| | Units/mg. Protein | Units/g. wet weight | Units/mg. Protein | Units/g. wet weight |
| Normal | 132±21 | 2·99±0·52 | 46±15 | 1250±353 |
| Fibroadenomata | 186±49 | 3·72±0·94 | 87±21 | 1890±406 |
| Fibrocystic | 290±62 | 4·73±1·32 | 60±4 | 920±116 |
| Carcinomata | 546±31 | 14·82±3·28 | 115±33 | 2610±368 |

| | ICDH | | PGDH | |
|---|---|---|---|---|
| | Units/mg. Protein | Units/g. wet weight | Units/mg. Protein | Units/g. wet weight |
| Normal | 10±3 | 230±62 | 2·3±0·6 | 51±10 |
| Fibroadenomata | 21±6 | 480±152 | 2·3±0·8 | 51±15 |
| Fibrocystic | 28±5 | 430±103 | 2·8±1·7 | 48±25 |
| Carcinomata | 41±7 | 1110±259 | 7·0±1·0 | 191±52 |

Mean ± S.E. of 5 samples in each group. Nucleases as μg. nucleic acid phosphorus solubilised/hour; dehydrogenases as mμM substrate transformed/min. at 25° C.; ADase as μM (mM) deaminated/hour/mg. protein (g. wet weight).

TABLE IV.—*Comparative Statistical Evaluation of Enzyme Data for Breast Tissue Supernatant*

| | Fibrocystic v Normal | | Carcinomata v Normal | | Carcinomata v Fibrocystic | |
|---|---|---|---|---|---|---|
| | t | P< | t | P< | t | P< |
| Alk. RNAase/mg. Protein | 1·05 | NS | 2·96 | 0·02 | 2·46 | 0·05 |
| Alk. RNAase/g. wet weight | 0·17 | NS | 3·92 | 0·005 | 3·94 | 0·005 |
| Acid RNAase/mg. Protein | 0·93 | NS | 3·42 | 0·01 | 3·79 | 0·01 |
| Acid RNAase/g. wet weight | 0·06 | NS | 4·86 | 0·005 | 4·29 | 0·005 |
| DNAase I/mg. Protein | 1·77 | NS | 0·13 | NS | 1·46 | NS |
| DNAase I/g. wet weight | 0·68 | NS | 0·44 | NS | 0·16 | NS |
| DNAase II/mg. Protein | 3·70 | 0·01 | 2·95 | 3·50 | 1·46 | NS |
| DNAase II/g. wet weight | 2·25 | NS | 0·02 | 0·01 | 2·64 | 0·05 |
| ADase/mg. Protein | 2·39 | 0·05 | 10·08 | 0·001 | 3·61 | 0·01 |
| ADase/g. wet weight | 1·16 | NS | 3·94 | 0·005 | 2·81 | 0·025 |
| LDH/mg. Protein | 0·83 | NS | 1·85 | NS | 1·70 | NS |
| LDH/g. wet weight | 0·91 | NS | 2·67 | 0·05 | 4·07 | 0·005 |
| ICDH/mg. Protein | 2·92 | 0·02 | 3·92 | 0·01 | 1·21 | NS |
| ICDH/g. wet weight | 0·91 | NS | 4·01 | 0·01 | 2·18 | NS |
| PGDH/mg. Protein | 1·59 | NS | 3·77 | 0·01 | 1·82 | NS |
| PGDH/g. wet weight | 1·52 | NS | 2·67 | 0·05 | 2·16 | NS |

Data analysed according to Student's t-test. NS = not significant.

*Enzyme activity of the particle fractions*

The technique used for assay of ADase activity is not suitable for application to turbid preparations and could not be used with this material. As with the protein results, the data have been compiled under " malignant " and " non-malignant " categories for the purpose of presentation in Table V. The particle fractions from 2 samples of " malignant " and 2 of " non-malignant " tissue were assayed for dehydrogenase activities. None of the 8 preparations so examined contained detectable activity for LDH, ICDH or PGDH. Consequently the data shown in Table V refer only to the nucleases. Because, relative to the other estimations, much more material was needed for DNAase assays, results for these enzymes were less frequently available.

The specific activities of all 4 nucleases were higher in the particle fractions from malignant samples, but because of the high scatter associated with a small number of results, statistical significance could not be demonstrated. As a consequence of the low particulate protein content of the " non-malignant " samples, these changes were magnified when enzyme activity was measured relative to tissue weight, and the differences between the alk. and acid RNAase content of the mitochondrial fractions were significant at the 0·1% (t = 5·52) and 0·5% (t = 4·29) levels respectively.

Only a very tentative comparison of the specific activities of the nucleases in the particulate fractions with those in the supernatant is justified by the present work. The activities of both RNAases are in the same range in all fractions of the tissues. There is a suggestion that, relative to the corresponding supernatant, the particle-bound DNAase I activity is greatly increased, while that of DNAase II is somewhat diminished, especially in the carcinomata.

TABLE V.—*Nuclease Activities of Breast Tissue Particulate Fractions*

| | Alk. RNAase | | Acid RNAase | |
|---|---|---|---|---|
| | Units/mg. Protein | Units/g. wet weight | Units/mg. Protein | Units/g. wet weight |
| Non-malignant Mitochondria | (5) 15·2± 4·7 | (5) 24·6±15·7 | (5) 5·9± 2·4 | (5) 6·4±2·1 |
| Malignant Mitochondria | (3) 83·1±41·5 | (3) 225·2±14·9 | (3) 39·2±15·1 | (3) 135·1±18·5 |
| Non-malignant Microsomes | (3) 17·0 | (3) 6·4 | (3) 4·1 | (3) 2·9 |
| Malignant Microsomes | (3) 68·4 | (3) 106·5 | (3) 26·9 | (3) 38·6 |

| | DNAase I | | DNAase II | |
|---|---|---|---|---|
| | Units/mg. Protein | Units/g. wet weight | Units/mg. Protein | Units/g. wet weight |
| Non-malignant Mitochondria | (2) 0·59 | (2) 0·13 | (2) 1·47 | (2) 0·31 |
| Malignant Mitochondria | (2) 1·03 | (2) 9·55 | (2) 4·14 | (2) 35·1 |
| Non-malignant Microsomes | (1) 1·01 | (1) 0·24 | (1) 1·56 | (1) 0·32 |
| Malignant Microsomes | (2) 1·81 | (2) 2·14 | (2) 5·78 | (2) 6·76 |

Mean values for each enzyme. S.E. given where relevant to statistical evaluation (see text). Number of samples in parenthesis. Activities as defined in legend to Table III.

### DISCUSSION

In the non-malignant samples of breast tissue, the activity of RNA-splitting enzymes was greater at pH 7·4 than at pH 5·6; the activity of DNAase II was greater than that of DNAase I; the ability of the samples to degrade RNA was greater than their DNA-splitting capacity; and the relative dehydrogenase activities were, in order of diminishing capacity, LDH, ICDH, and PGDH. All these properties were previously found in non-malignant samples of the human cervix uteri (Goldberg and Pitts, 1966; Ayre and Goldberg, 1966), and it is possible that this pattern is common to all human epithelial tissues.

### *The nature of the enzyme activities*

The distribution of RNAase activities tentatively suggests certain differences. In the non-malignant tissues supernatant acid RNAase activity was half that of the corresponding alk. RNAase, while the particle-bound acid RNAase activity was $\frac{1}{3}$ that of the corresponding alk. RNAase. In the malignant tissues, these ratios were $\frac{2}{3}$ and $\frac{2}{5}$ respectively. There is thus some evidence for a relative decrease of acid RNAase in the particles. Since this enzyme is located in lysosomes in rat mammary gland (Greenbaum, Slater and Wang, 1960; Slater, 1961) the rupture of these particles by freezing and thawing with solubilisation of acid RNAase may be presumed. The alk. RNAase of rat mammary gland has been reported to be of mitochondrial origin (Slater, 1961). Freezing and thawing might be expected to have a less pronounced effect on such particles, and this would account for the higher relative particle-bound activity of this enzyme in the present material.

DNAase I specific activity was greater in the particle fractions, while that of DNAase II was higher in the supernatant. There seems little doubt that these activities are due to different enzyme components. Although DNAase II activity is associated with lysosomes in most tissues (De Duve, Wattiaux and Baudhuin, 1962), this does not appear to be so in rat mammary gland (Greenbaum *et al.*, 1960), and it is probable that likewise in the corresponding human tissue the bulk of the enzyme is of supernatant origin. We have been unable to detect dehydrogenase activity other than in the supernatant fraction of human mammary tissue.

### *Relationship between enzyme changes and tissue pathology*

For all the enzymes studied, the breast tissues, with few exceptions, showed a general increase in supernatant activity in the following order: normal, fibroadenomata, fibrocystic, and carcinoma. A precise evaluation of the extent to which these changes represent genuine increases in enzyme content of the acinar cells is not possible. At least two other cell types were present in the material: fat cells and connective tissue cells. The former were relatively minor constituents, and while the latter were abundant in all samples, it was our impression from histological examination that the amount of fibrous tissue bore an inverse relationship to the soluble protein content as estimated by chemical analysis. Indeed the well-known insolubility of collagen and of connective tissue components in general (Eastoe and Courts, 1963) renders it probable that the bulk of the soluble protein was derived from the acinar cells, and that the specific enzyme activities offer a useful guide to the enzyme content of the epithelial cells.

If this reasoning is correct, we may state that increased nuclease, adenosine deaminase and dehydrogenase content of the human mammary epithelial cell takes place during the transition from the normal state to hyperplastic and neoplastic states, this increase being greatest when the stage of invasive carcinoma is attained. Although scanty, the data on particle-bound nuclease activity support the idea that we are dealing with genuine differences between cells of epithelial type, since it is likely that contamination of this material by non-epithelial elements would be even less than that possible for the corresponding supernatants.

The biochemical changes found in breast cancers were similar to those that distinguish malignant from non-malignant cervix uteri (Goldberg and Pitts, 1966; Ayre and Goldberg, 1966) and may be characteristic of human epithelial cancers. The increased levels of nucleic acid-splitting enzymes may form part of the invasive apparatus of the cancer cells. Many reports testify to the high content of nucleases that develops in cells subjected to viral invasion (Wormser and Pardee, 1957; Korn and Weissbach, 1963; Keir and Gold, 1963; Russell *et al.*, 1964). A correlation between virulence and DNAase activity has been established for certain bacteria (Jacobs, Willis and Goodburn, 1963); and impairment of various metabolic functions, especially protein synthesis and oxidative phosphorylation, has been demonstrated after addition of pancreatic RNAase to cells growing in culture (Firket, Chevremont-Comhaire and Chevremont, 1955; Groth, 1956; Hanson, 1959; Jeener, 1959*a*, 1959*b*; Jeener, Dupont-Mairesse and Vansanten, 1960). The pronounced increase in the NADP-linked dehydrogenases, in contrast to the relatively minor change in activity of the NAD—linked LDH, is compatible with the suggestion of Potter (1956) that cancers may have increased capacity for generating reduced NADP required for synthesis of thymidine, which step is probably rate-limiting for DNA synthesis.

It is interesting that the fibroadenomata, which do not give rise to cancers, showed no significant differences when compared with normal breast tissue, while the samples removed from breasts with fibrocystic disease differed from the normal in several respects; these changes were in the same direction as those found in the cancer tissue but of less pronounced degree. From the standpoint of this investigation, fibrocystic disease would seem to occupy an enzymological position midway between normal and malignant tissue. This would fit with its status as a pre-malignant lesion (Willis, 1960; Sandison, 1962; Davis, Simons and Davis, 1964). It may be noted that all the samples were the site of epithelial hyperplasia, although the proportion of fibrous tissue present was greater than that encountered in the other conditions, thus accounting for the low soluble protein content of the tissues.

Since Sandison (1962) considers epithelial hyperplasia of the type seen in all 5 samples with fibrocystic disease to be the key pathological change in the involuting breast, it is interesting that forced mammary involution in the rat is accompanied by an early rise in activity of acid hydrolases, including RNAase (Greenbaum, Slater and Wang, 1965), while the catabolism of glucose by the various pathways is reduced (Greenbaum and Darby, 1964), and thus it is unlikely that the activities of the enzymes concerned could be raised. This pattern of increased nuclease activity and unchanged dehydrogenase activity is precisely that seen in the present fibrocystic material.

*Present work in relation to previous reports*

Our results in human mammary cancer contrast with those of Daoust and Amano (1963) who reported decreased RNAase and DNAase activity in material studied by histochemical techniques. These findings have been criticised by Roth (1963), but examination of rat liver tumours by an immunofluorescence technique supports the conclusions derived from histochemical analysis of RNAase in this tissue (Gordon and Myers, 1966). It must be pointed out, however, that changes in the characteristics of these enzymes can occur during malignancy, as reported by Colter, Kuhn and Ellem (1961) for RNAases of mouse ascites tumours, and by Georgatsos and Symeonidis (1965) for DNAases of mouse mammary tumours. It is not unreasonable to suggest that the above techniques are, in their present state of development, not yet capable of providing the sensitivity and precision possessed by the conventional methods used in this study.

Measurements of dehydrogenases have been reported in various rodent tumours. The activities of 3 NADP-linked enzymes, including ICDH, were increased in experimental rat mammary tumours (Hilf *et al.*, 1965), but changes in ICDH were found less frequently in response to endocrine ablation or hormone treatment (Hilf *et al.*, 1966). Increased production of lactate from glucose takes place in the Barrett mammary adenocarcinoma of mice (Abraham and Chaikoff, 1965). This tumour has a higher content of LDH and a lower content of hexose monophosphate shunt enzymes than normal mouse mammary tissue, whereas pre-neoplastic alveolar nodules of breast have lower LDH than normal and higher levels of hexose monophosphate shunt enzymes than the cancers (Kopelovich *et al.*, 1966). Rat mammary tumours induced by 3-methylcholanthrene had increased LDH and ICDH relative to wet weight and protein content, but of the 2, only LDH was increased relative to DNA-phosphorus (Hershey *et al.*, 1966). There is thus ample precedence for increased ICDH in mammary tumours, but the relative insensitivity of LDH and the significant increase in PGDH activity in the human tumours contrast with the above reports on rodent tumours.

The paucity of cytoplasmic particles found in the non-malignant samples in this study is consistent with several reports based on electron microscopy. Normal resting human breast tissue contains only 5–13 mitochondria per cell and is essentially devoid of rough-surfaced ergastoplasm (Waugh and Van der Hoeven, 1962). The fine structure of the cells in fibrocystic disease resembles that of normal breast cells (Wellings and Roberts, 1963). Breast cancers show considerable variation in their ultrastructure, but in general it would appear that medullary carcinomas and mucin-producing carcinomas have abundant mitochondria and well-developed endoplasmic reticulum, whereas these structures are less well represented in scirrhous cancers (Wellings and Roberts, 1963; Murad and Scarpelli, 1965; Tellem *et al.*, 1966). A direct quantitative comparison between the cytoplasmic particle content of normal and malignant human breast does not seem to have been made. The question of whether the differences in particle protein content between malignant and non-malignant breast tissue as recorded in the present work represent technical difficulties or genuine differences in cellular ultrastructure can only be answered by direct counting; such a technique has been successfully applied to the rat mammary gland, and has elucidated certain aspects of lactation and involution in this organ (Greenbaum and Slater, 1957).

SUMMARY

(1) The activities of alkaline and acid ribonuclease, deoxyribonuclease II (DNAase II) and adenosine deaminase (ADase) were greater relative to wet weight and protein content in the supernatant fraction of human breast cancers as compared with the corresponding activities in non-malignant human breast tissue. DNAase I activity was of the same order in the supernatant fractions of malignant and non-malignant samples.

(2) The activities of lactate dehydrogenase, isocitrate dehydrogenase (ICDH) and phosphogluconate dehydrogenase were higher, relative to wet weight, in the supernatant fraction of the cancers than the corresponding activities in normal breast tissue. The latter 2 activities were also higher in the cancers relative to protein content. None of these enzymes could be detected in cytoplasmic particulate fractions prepared from malignant and non-malignant breast tissue.

(3) The activities of all these enzymes in breast fibroadenomata were, for the most part, a little higher than those of the normal breast tissue, but none of these differences were significant. By contrast, tissue from cases of fibrocystic disease had levels which were generally midway between those of the normal and cancer tissues, and the specific activities of DNAase II, ADase and ICDH relative to protein were significantly higher than the normal levels.

(4) The supernatant protein content of the samples showed an inverse relationship to the proportion of fibrous tissue present, the value for the fibrocystic samples being significantly below the normal level.

(5) Cytoplasmic particulate fractions were prepared from a small series of malignant and non-malignant tissues. The nuclease activities of the malignant samples exceeded those of the non-malignant samples, and the protein content of the former exceeded that of the latter.

We are indebted to Dr. E. B. Hendry who allowed generous facilities within his department, Professor D. F. Cappell for the use of certain apparatus, and Professor J. N. Davidson, F.R.S. and Dr. R. Y. Thomson for their advice and guidance. We should also like to thank our surgical colleagues, and in particular Mr. R. Shields, who provided the specimens used in this investigation.

REFERENCES

Abraham, S. and Chaikoff, I. L.—(1965) *Cancer Res.*, **25**, 647.
Ayre, H. A. and Goldberg, D. M.—(1966) *Br. J. Cancer*, **20**, 743.
Colter, J. S., Kuhn, J. and Ellem, K. A. O.—(1961) *Cancer Res.*, **21**, 48.
Daoust, R. and Amano, H.—(1963) *Cancer Res.*, **23**, 131.
Davis, H. H., Simons, M. and Davis, J. B.—(1964) *Cancer, N.Y.*, **17**, 957.
De Duve, C., Wattiaux, R. and Baudhuin, P.—(1962) *Adv. Enzymol.*, **24**, 291.
Eastoe, J. E. and Courts, A.—(1963) 'Practical Analytical Methods for Connective Tissue Proteins'. London (E. and F. N. Spon Ltd.).
Firket, H., Chevremont-Comhaire, S. and Chevremont, M.—(1955) *Nature, Lond.*, **176**, 1075.
Georgatsos, J. G. and Symeonidis, A.—(1965) *Nature, Lond.*, **206**, 1362.
Goldberg, D. M. and Pitts, J. F.—(1966) *Br. J. Cancer*, **20**, 729.
Gordon, J. and Myers, J.—(1966) *Biochim. biophys. Acta*, **113**, 187.
Greenbaum, A. L. and Darby, F. J.—(1964) *Biochem. J.*, **91**, 317.
Greenbaum, A. L. and Slater, T. F.—(1957) *Biochem. J.*, **66**, 161.

GREENBAUM, A. L., SLATER, T. F. AND WANG, D. Y.—(1960) *Nature, Lond.*, **188**, 138—(1965) *Biochem. J.*, **97**, 518.
GROTH, D. P.—(1956) *Biochim. biophys. Acta*, **21**, 18.
HANSON, J. B.—(1959) *J. biol. chem.*, **234**, 1303.
HERSHEY, F. B., JOHNSTON, G., MURPHY, S. M. AND SCHMITT, M.—(1966) *Cancer Res.*, **26**, 265.
HILF, R., MICHEL, I., BELL, C., FREEMAN, J. J. AND BORMAN, A.—(1965) *Cancer Res.*, **25**, 286.
HILF, R., MICHEL, I., BELL, C. AND CARRINGTON, M. J.—(1966) *Cancer Res.*, **26**, 1365.
JACOBS, S. I., WILLIS, A. T. AND GOODBURN, G. M.—(1963) *Nature, Lond.*, **200**, 709.
JEENER, R.—(1959*a*) *Biochim. biophys. Acta*, **32**, 99.—(1959*b*) *Biochim. biophys. Acta*, **32**, 106.
JEENER, R., DUPONT-MAIRESSE, N. AND VANSANTEN, G.—(1960) *Biochim. biophys. Acta*, **45**, 606.
KEIR, H. M. AND GOLD, E.—(1963) *Biochim. biophys. Acta*, **72**, 263.
KOPELOVICH, L., ABRAHAM, S., MCGRATH, H., DEOME, K. B. AND CHAIKOFF, I. L.—(1966) *Cancer Res.*, **26** 1534.
KORN, D. AND WEISSBACH, A.—(1963) *J. biol. chem.*, **238**, 3390.
MURAD, T. M. AND SCARPELLI, D. G.—(1965) *Lab. Invest.*, **14**, 595.
POTTER, V. R.—(1956) *Cancer Res.*, **16**, 658.
ROTH, J. S.—(1963) *Cancer Res.*, **23**, 657.
RUSSELL, W. C., GOLD, E., KEIR, H. M., OMURA, H., WATSON, D. H. AND WILDY, P.—(1964) *Virology*, **22**, 103.
SANDISON, A. T.—(1962) ' An Autopsy Study of the Adult Human Breast '. (*Natn. Cancer Inst. Monogr.*, No. 8).
SLATER, T. F.—(1961) *Biochem. J.*, **78**, 500.
TELLEM, M., NEDWICH, A., AMENTA, P. S. AND IMBRIGLIA, J. E.—(1966) *Cancer, N Y.*, **19**, 573.
WAUGH, D. AND VAN DER HOEVEN, E.—(1962) *Lab. Invest.*, **11**, 220.
WELLINGS, S. R. AND ROBERTS, P.—(1963) *J. natn. Cancer Inst.*, **30**, 269.
WILLIS, R. A.—(1960) ' Pathology of Tumours '. London (Butterworth).
WORMSER, E. H. AND PARDEE, A. B.—(1957) *Virology*, **3**, 76.